# Does the Association between Preschool Media Use and Language Difficulties at School Entry Vary by First Language of the Child and Parental Education?

**DOI:** 10.3390/children10121848

**Published:** 2023-11-25

**Authors:** Chiara Maria Hammrich, Simon Götz, Monika Daseking, Simone Weyers

**Affiliations:** 1 Institute of Medical Sociology, Centre for Health and Society, Medical Faculty and University Hospital Düsseldorf, Heinrich Heine University Düsseldorf, Germany, Moorenstrasse 5, 40225 Duesseldorf, Germany; chiara.hammrich@uni-duesseldorf.de (C.M.H.); simon.goetz@uni-duesseldorf.de (S.G.); 2 Educational Psychology, Helmut Schmidt University/University of the Federal Armed Forces Hamburg, Holstenhofweg 85, 22043 Hamburg, Germany; dasekinm@hsu-hh.de

**Keywords:** language difficulties, school entry examination, media use, preschool children, first language, parental education

## Abstract

Background: Both media use and social background affect children’s language development. The aim of this study was to explore the association between media use and different aspects of language difficulties (grammar, auditory memory, articulation) and social background (first language (FL), parental education (PE)) in more detail. Methods: We analyzed data from 4015 children from the 2013/14 school entry examination in a Western German city. Media use, FL, and PE were assessed by social history, and language difficulties by sociopediatric screening. We calculated Prevalence Ratios with a 95% Confidence Interval for language difficulties by media use and FL/PE; compared models with and without interaction terms; and estimated the predicted prevalence of language difficulties by media use and FL/PE. Results: Children with non-German FL/low PE had a higher media use and more language difficulties. However, the gradual association between media use and grammatical abilities/recommendation of training was more pronounced in children with German FL and medium/high PE. e.g., especially in the preposition subtest. Conclusions: The association between media use and language difficulties varies regarding different aspects of social background and language difficulties. Still, extensive media use is linked with language difficulties for all children. The media competence of young families remains crucial in times of digitalization.

## 1. Introduction

Impairments of language development are among the most frequent developmental disorders in young children [1]. German health insurance data showed that one third of five-year-old children were diagnosed with language development disorders as coded in the International Classification of Diseases and Health Related Problems (ICD-10) [2]. In the German school entry examination, 28 to 30% of children did not have age-based language development [3]. The survey data showed that the prevalence of logopedic intervention in three-to-five-year-old children amounts to 15% [4].

Language acquisition is an important developmental task in childhood. Language is a central means of expression and serves to exchange information, thoughts, and feelings. It is embedded in the overall development of a child as it interacts with other areas such as sensory, motor, social, emotional, and cognitive development [1]. On the one hand, language supports development in other domains; on the other hand, there are correlations between language development disorders and deficits in other developmental domains such as motor development [5].

Early language difficulties have consequences for school performance, for instance, in terms of reading, writing, and arithmetic [6]. Especially for reading literacy, language development is a key predictor [7]. Children with early delays in language development are at increased risk of reading problems later in life [8], especially children from low socio-economic status (SES) families [9]. Language development also plays an important role in children’s psychosocial adjustment in family or school [10]. Cohen found a correlation between language development and social-emotional development [11]. Language performance also shows connections to behavior control and emotion regulation [12]. In children with early language difficulties, i.e., also with a language development disorder, language continues to develop. Children learn and use compensatory strategies, which means that spontaneous speech is no longer conspicuous later, but long-term developmental problems can still usually be mapped. This refers especially to the domain of pragmatics and becomes clear in adolescence or adulthood when telling stories or using figurative language [13].

Despite various other predictors such as a familial disposition, neurological maturational disorders, and prenatal influences of neurotoxic substances [14,15], media use has turned out to be a behavioral risk factor for developing language difficulties. Media refers to a comprehensive and heterogeneous set of technologies and to the content transmitted through these technologies [16]. Media use in this context usually means the use of screen-based media (especially TV, video game playing, and computer use). Ennemoser et al. found an association between higher media use and language difficulties at the beginning of primary school [17]. More specifically, a TV in the child’s bedroom meant a risk of developing language disorders [18]. On the other hand, there are also study results that prove a positive effect of guided media consumption, for example, in schools or daycare centers, on cognitive development [19]. Age, the type of medium, the content, and the social context in which it is used could play an important role in media use: In children under the age of 2, television consumption has predominantly negative effects, particularly on language and executive functions. For preschool children, both positive and negative effects can be observed, with educational television having a positive effect on cognitive development [20]. New evidence confirms higher preschool media use as a risk factor [3,21]. Children with a high screen time had a lower performance in cognition, language, and social-emotional skills than children with normal use [22].

The development of language skills depends on the environment of the child, too. This especially applies to vocabulary but also grammar [23]. The family learning environment can, e.g., differ regarding (daily) reading aloud and the spoken language at home as well as in resources such as household income, level of education, or parental employment status. Children receive different stimulation for the development of their language skills right from the beginning. A higher prevalence of language difficulties has been observed in children from low SES families and those with a migration background. Children from low SES families had findings of language development more often compared to those with a high SES. Similar results were found regarding migration background [24]. In a school entry examination, children from low SES families were found to be at higher risk of language difficulties compared to better-off children, whereas the results regarding migration background were mixed [3]. Also, children from low SES families more often made use of logopedic therapy [4].

However, little is yet known about the interaction of preschool media use and first language of the child (FL)/parental education (PE) with language difficulties at school entry. For instance, extensive media use could add to a lack of linguistic stimulation in children from low SES families. On the other hand, media use could compensate a foreign FL in children with a migration background. Therefore, we aim to answer the following questions: Does the association between preschool media use and language difficulties vary regarding different aspects of language? Does the association between preschool media use and language difficulties vary regarding the FL of the child and PE?

## 2. Materials and Methods

### 2.1. Participants and Data

This cross-sectional study is based on the mandatory school entry examination of 4015 children, conducted by municipal health authorities in Rhein-Kreis Neuss between 2013 and 2014. Children were examined 6 to 12 months before school entry and variables were assessed by school doctors using standardized anamnesis: Daily time of the child’s media use (independent variable) was categorized into 0–<30 min/30–<60 min/1–2 h/>2 h. First language (FL, interaction variable) was dichotomized into German language mainly spoken during first years of life vs. other language. Parental education (PE, interaction variable) included school and vocational education of both parents [25] and was dichotomized into medium and high PE vs. low PE (both parents, at most, had a secondary school certificate without vocational education).

Language development was assessed by sociopediatric development screening for school entry examinations (SOPESS), including four linguistic subtests (preposition, plural formation, pseudowords, articulation, dependent variables). SOPESS is a standardized and highly economized screening with few items assessing the child’s requirements for learning at school and trying to detect possible risk factors for a healthy development. Its aim is to differentiate between children with and without conspicuousness to carry out further diagnostics and provide specific training if needed [26].

As a common approach to screenings, test scores below the 10th percentile were considered as conspicuous, and between the 10th and 25th percentile as a marginal finding [27]. We merged these two categories vs. children without findings to receive a dichotomous variable (findings yes/no). Additionally, the overall finding (dependent variable) in language development was coded according to the Bielefeld model [28] and dichotomized with categories X (need for surveillance), A (first or further referral necessary), B (already in therapy), and D (considerable, not only temporary impairment) vs. K (no findings). 0 (examination could not be carried out) was coded as a missing value. Finally, recommendation of language training (dependent variable) was added (yes/no). Age and gender were included as covariates.

Generally, all children with school entry in 2014 in the given community were included in our study. The following children were excluded: Children with missing information in the dataset (*n* = 671). Children with health problems likely to affect the development of language (hearing disorder, neuromotor disorder, learning disorder, mental disability, behavioral conspicuousness, conspicuous language development at the age of 24 months (less than 50 words)) and/or children already receiving logopedic/speech therapy (*n* = 1084). Our purpose was to exclude as many already linguistically conspicuous children as possible to improve the quality of our study results and reduce the effect of vice-versa associations between media use and language deficits. The final sample comprised 2260 children (56% of the original total sample).

The study design was conducted in accordance with the Declaration of Helsinki and approved by the Ethics Committee of the Duesseldorf Medical Faculty (study nr. 5846R).

### 2.2. Data Analysis

To analyze if language difficulties are associated with media use and FL/PE, frequencies of the six indicators of language development were described according to media use, stratified by FL/PE. To test if differences by FL or PE are significant, we used chi-square tests. In a next step, we estimated a series of multivariable regression models by Poisson regressions [29]. We calculated Prevalence Ratios (PRs) with a 95% Confidence Interval (CI), stratified for FL and PE and adjusted for age, gender, and either FL or PE. To assess if the association between media use and language difficulties varies by FL or PE, we added interaction terms and compared models with and without interaction terms, using Wald test. Finally, we estimated the predicted prevalence (%) of language difficulties by media use and either FL or PE (based on multivariable regression models, adjusted for age, gender, and either FL or PE). We conducted all analyses using Stata 14.

## 3. Results

Table 1 displays the sample characteristics: 6.6% of the children had an elevated media use of >2 h (with marked differences according to first language (FL) and parental education (PE): 3.7% in children with German FL vs. 18% in children with non-German FL, *p* = 0.000; 4.8% in medium/high PE children vs. 18% in low PE children, *p* = 0.000). A total of 18% had findings in their overall language development. The subtest with the highest number of findings was articulation (17%), and 5.8% of the children received a recommendation of language training. The mean age of the population was 5.97 years with a 0.35 standard deviation.

**Figure 1:**
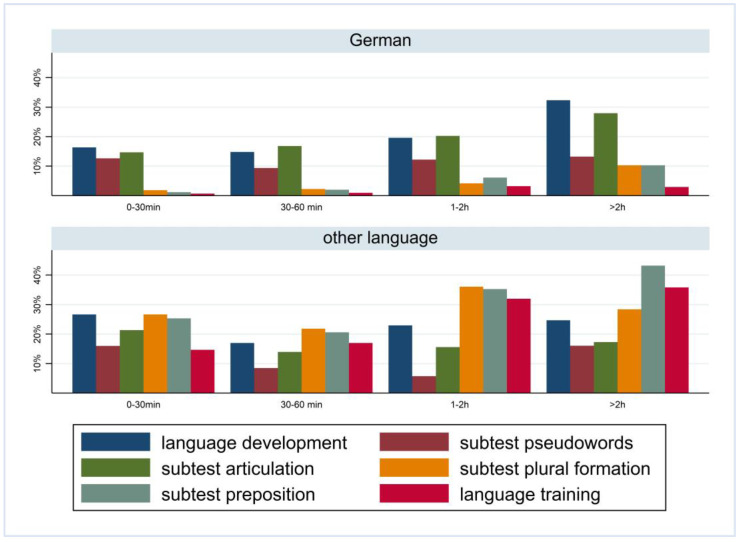
Prevalence of findings in language development by media use and first language.

**Figure 2:**
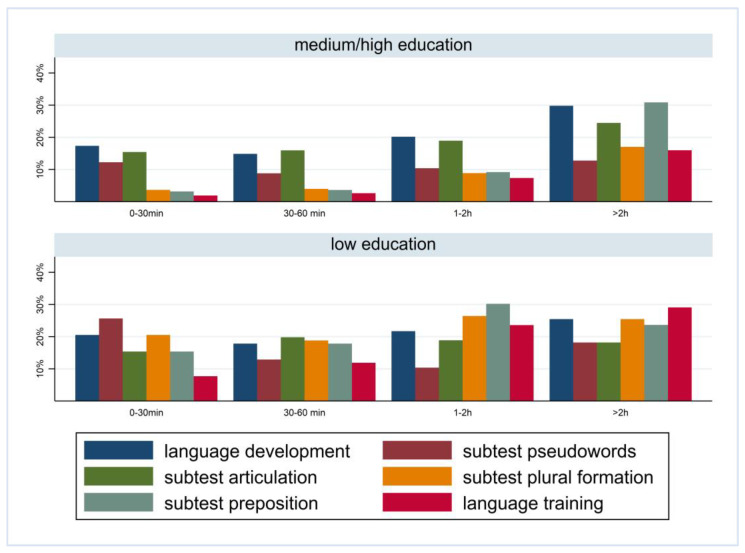
Prevalence of findings in language development by media use and parental education.

Figure 1 and Figure 2 show the prevalence of the overall findings and recommendation of language training according to daily media use and stratified by FL and PE. In children with German FL, we observed an increase in most indicators of language difficulties with increasing media use. In children with non-German FL, the pattern is mixed with an increase in plural formation, preposition, and training recommendation. The results are comparable regarding PE where children with medium/high PE had lower percentages of language difficulties and those increase with media use. Again, a mixed pattern was observed in children with lower PE.

**Figure 3:**
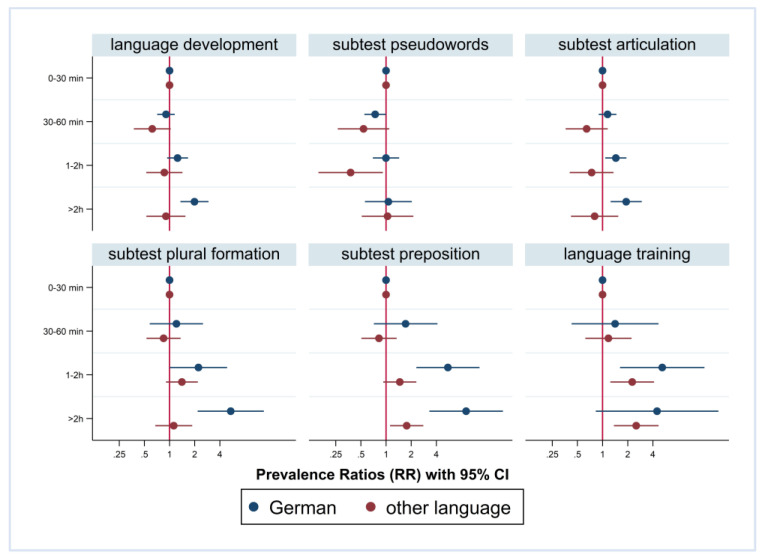
Prevalence Ratios of findings in language development and recommendation of training; by media use and stratified by first language; adjusted for parental education, age, and gender.

**Figure 4:**
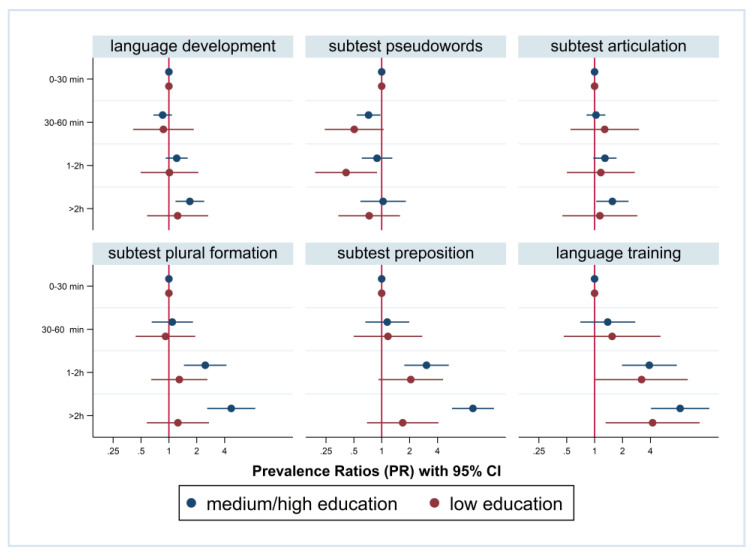
Prevalence Ratios of findings in language development and recommendation of training; by media use and stratified by parental education.

Figure 3 and Figure 4 show the Prevalence Ratios (PRs) with a 95% CI for the findings in the six indicators of language development by media use, stratified by FL and PE. Furthermore, we included interaction terms for media use and FL/PE, and children with a daily media use of 0–30 min are the reference category. Higher media use was associated with an increased risk of findings in language development. However, in children with German FL and in children with medium/high PE, the association was more pronounced. For instance, regarding the preposition subtest, the PR in children with German FL with a daily media use of more than 2 h was 9.08-fold compared to the reference group with up to 30 min of media use (CI 3.29; 25.02). In children with non-German FL, the PR was 1.77 (CI 1.12; 2.79). The difference according to FL was significant for preposition and plural formation but not for the other four outcomes.

Again, the results are comparable regarding PE. For instance, regarding preposition, the PR in children from medium/high PE families and daily media use of more than two hours was 9.67-fold compared to the reference group with up to 30 min of media use (CI 5.74; 16.30). In children from low PE families, the PR was 1.69 (CI 0.69; 4.10). Again, the difference according to PE was significant for preposition and plural formation but not for the other four outcomes.

**Figure 5:**
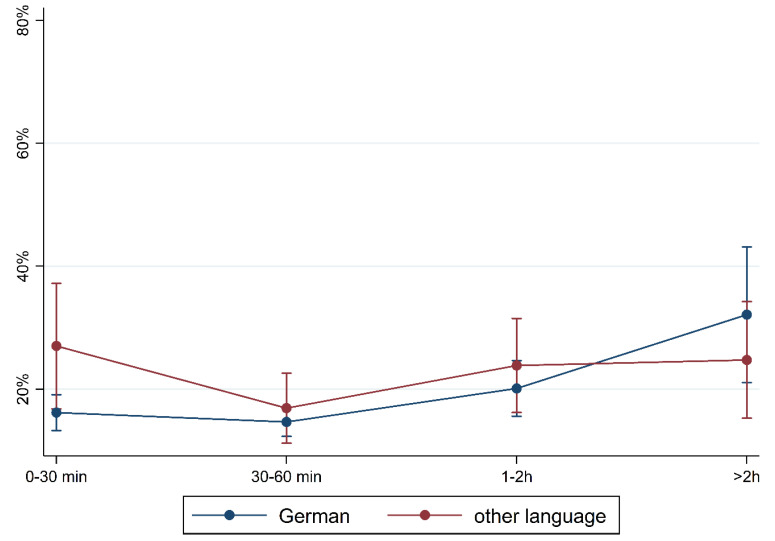
Predicted prevalence of overall findings of language development by media use and first language; adjusted for age, gender, and parental education.

**Figure 6:**
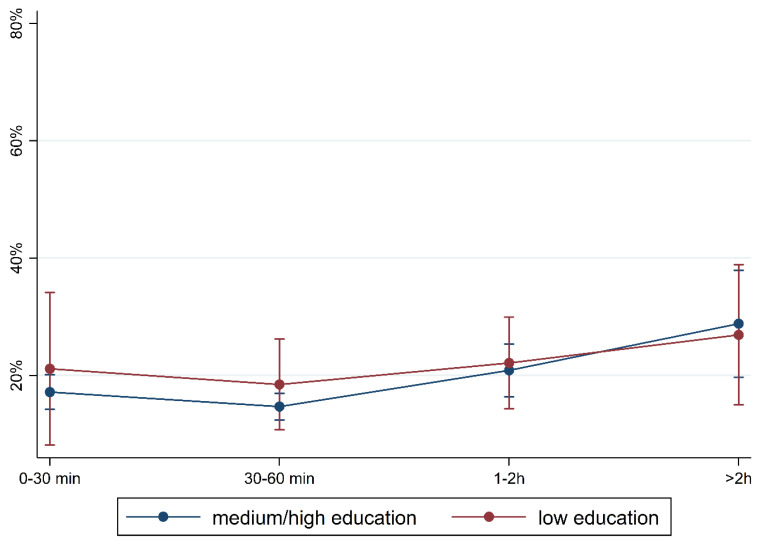
Predicted prevalence of overall findings of language development by media use and parental education; adjusted for age, gender, and first language.

**Figure 7:**
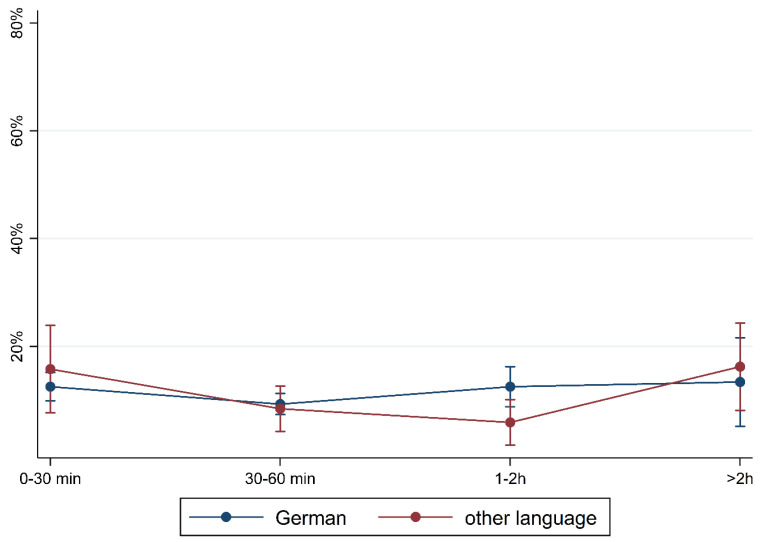
Predicted prevalence of findings in pseudowords subtest by media use and first language; adjusted for age, gender, and parental education.

**Figure 8:**
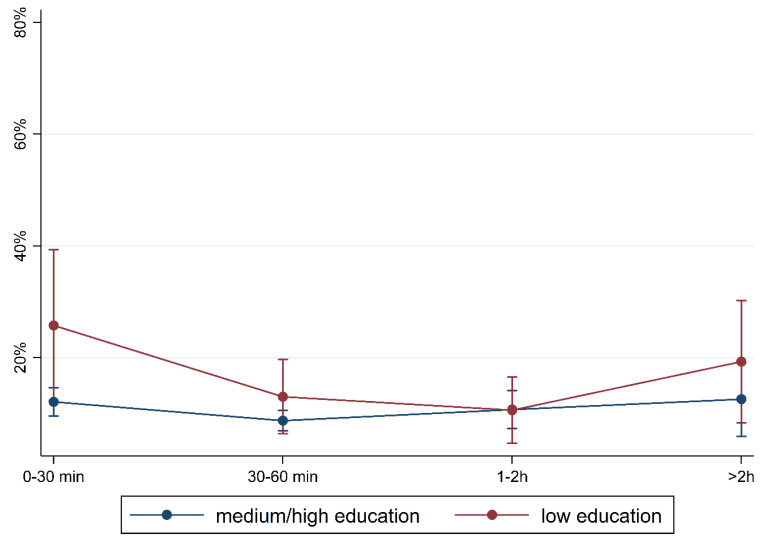
Predicted prevalence of findings in pseudowords subtest by media use and parental education; adjusted for age, gender, and first language.

**Figure 9:**
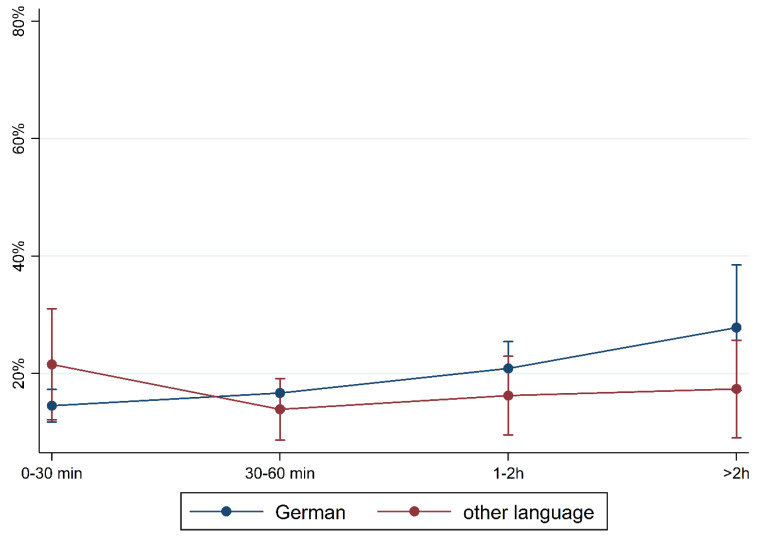
Predicted prevalence of findings in articulation subtest by media use and first language; adjusted for age, gender, and parental education.

**Figure 10:**
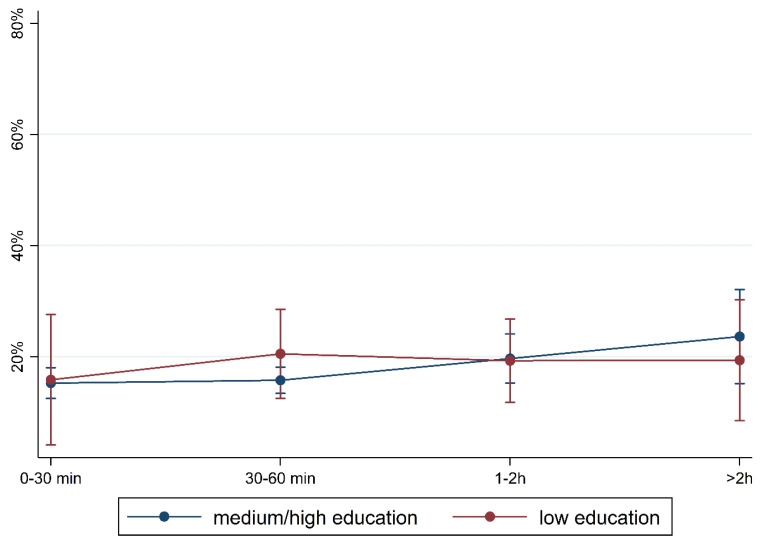
Predicted prevalence of findings in articulation subtest by media use and parental education; adjusted for age, gender, and first language.

**Figure 11:**
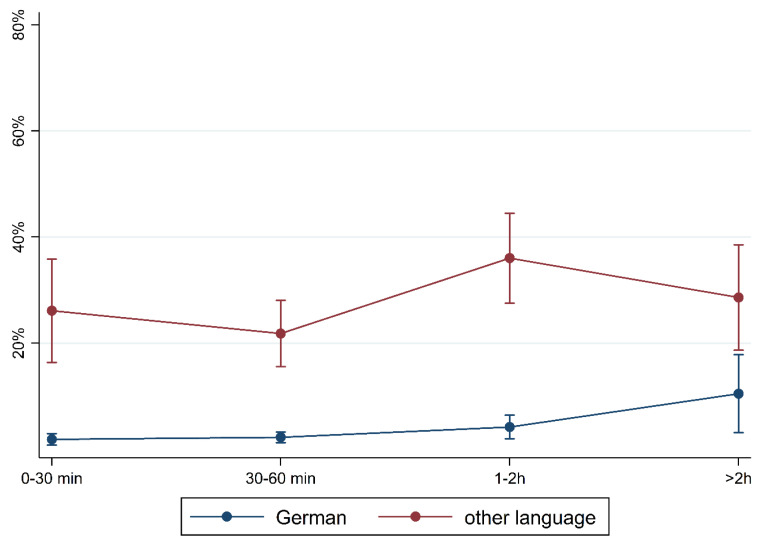
Predicted prevalence of findings in plural formation subtest by media use and first language; adjusted for age, gender, and parental education.

**Figure 12:**
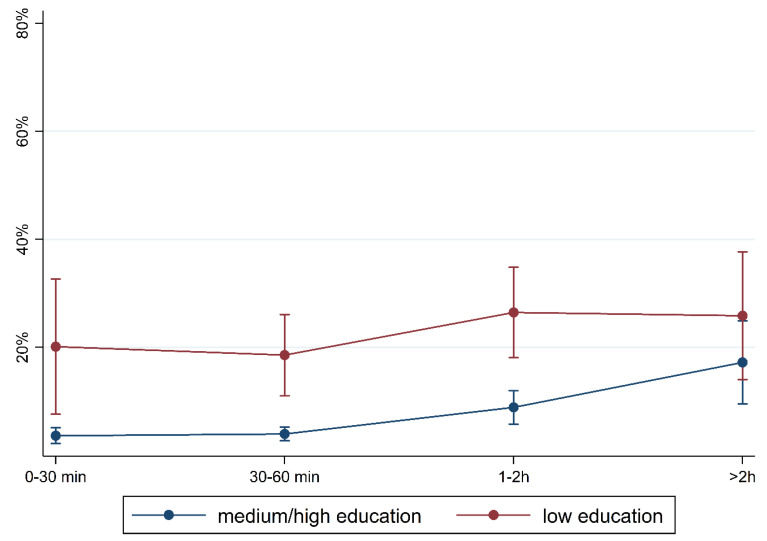
Predicted prevalence of findings in plural formation subtest by media use and parental education; adjusted for age, gender, and first language.

**Figure 13:**
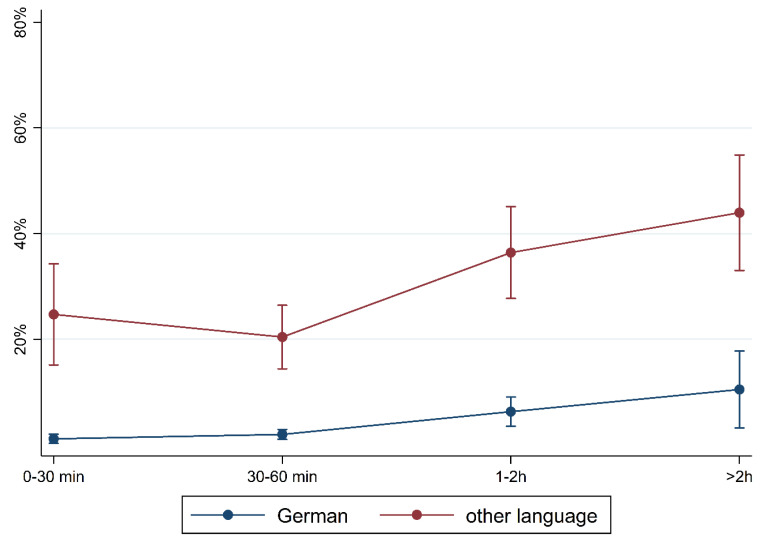
Predicted prevalence of findings in preposition subtest by media use and first language; adjusted for age, gender, and parental education.

**Figure 14:**
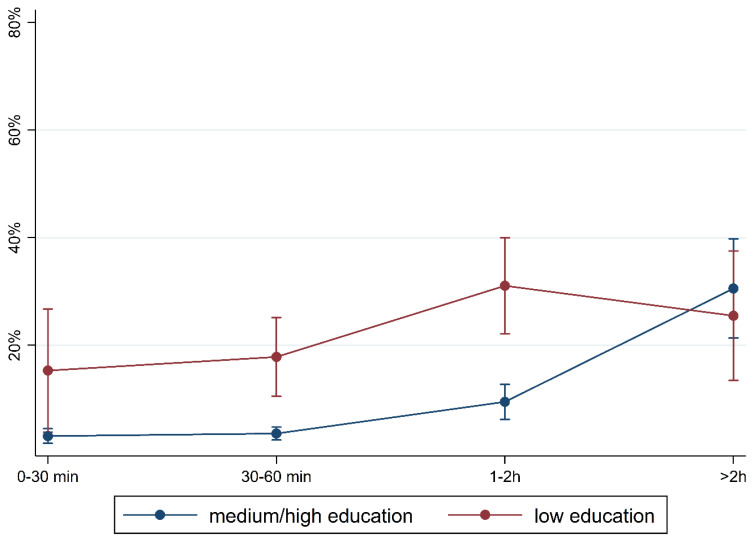
Predicted prevalence of findings in preposition subtest by media use and parental education; adjusted for age, gender, and first language.

**Figure 15:**
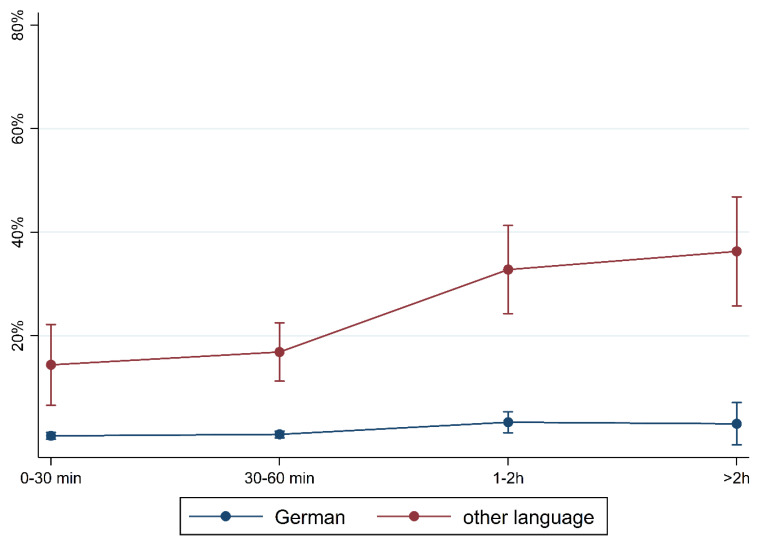
Predicted prevalence of recommendation of training by media use and first language; adjusted for age, gender, and parental education.

**Figure 16:**
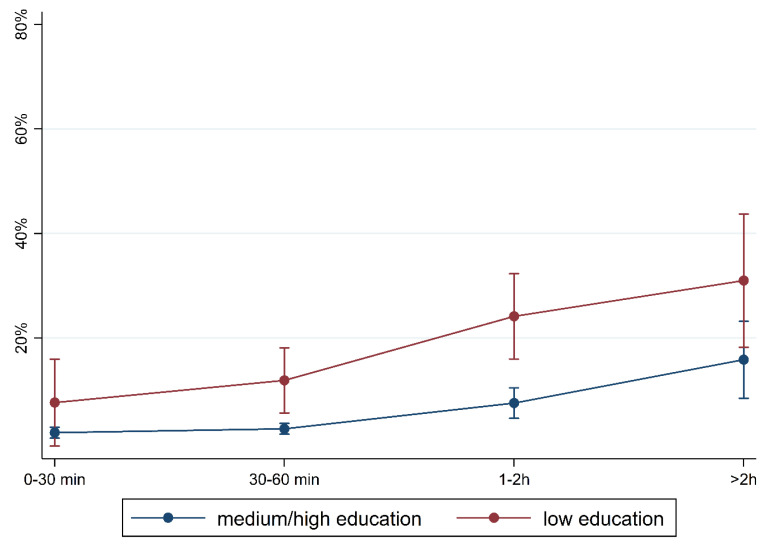
Predicted prevalence of recommendation of training by media use and parental education; adjusted for age, gender, and first language.

To illustrate the differences according to social background more clearly, Figure 5, Figure 6, Figure 7, Figure 8, Figure 9, Figure 10, Figure 11, Figure 12, Figure 13, Figure 14, Figure 15 and Figure 16 show the predicted prevalence of language difficulties by media use, stratified by FL/PE. The results were mixed. We did not observe a significantly higher prevalence in children with non-German FL and with low PE regarding overall language development (Figure 5 and Figure 6), pseudowords (Figure 7 and Figure 8), and articulation (Figure 9 and Figure 10). However, we observed a significantly higher prevalence in children with non-German FL and with low PE regarding plural formation (Figure 11 and Figure 12), preposition (Figure 13 and Figure 14), and recommendation of language training (Figure 15 and Figure 16).

## 4. Discussion

The aim of this study was to answer the following questions: Does the association between preschool media use and language difficulties vary regarding different aspects of language? Does the association between preschool media use and language difficulties vary regarding the FL of the child and PE?

There is a gradual association between media use and some aspects of language difficulties, specifically regarding grammatical abilities. Also, while children from vulnerable families use media more often, the association between preschool media use and language difficulties is more pronounced in better-off children.

To explain these results, it must be considered that preposition and plural formation represent grammatical ability tasks, while pseudowords measures auditory memory performance and articulation refers to the ability to pronounce sounds correctly. Children with non-German FL tend to perform even better in articulation and auditory working memory than children with German FL [27]. On average, children growing up multilingual were able to repeat more syllables and made fewer articulation errors than children in the comparison sample. This finding could be explained by better-developed executive functions in multilingualism [30].

Regarding the role of media, it could be hypothesized that, on the one hand, children with non-German FL may profit from higher media use. Linebarger and Walker have shown that media use makes children familiar with language-specific vocabulary and pronunciation [31]. This could explain why children with non-German FL in our study received similar or even better results in pseudowords and articulation compared to the reference group of children with German as FL. On the other hand, this effect does not show in preposition and plural formation examining grammatical abilities. As those are practiced by social interaction, high media use cannot replace that [32] and therefore apparently cannot compensate non-German FL in general.

Language development takes place in an interactive setting; the use of visual media is predominantly non-interactive. High media use deprives children of important experiences in the language learning context, not only in terms of learning vocabulary and grammar, but also in terms of the appropriate use of language. Therefore, we propose to distinguish between linguistic and visual media when asking questions about the (beneficial or harmful) effects of media. As a result of this differentiation, studies show that when using visual media, only the information from the pictures is used to be entertained, without it being necessary to process the associated language information at the same time [33].

Studies show that children growing up multilingually consume more image-focused visual media (like cartoons or movies) than children growing up monolingually but avoid language-focused media like audio plays/dramas [34]. Irrespective of the children’s FL, it is evident that children with deficits in pragmatic-communicative skills (as an aspect of language competence, e.g., appropriate and rules-based use of language in social context) are less likely to use language-enhancing media [35].

Our study has also shown that, in children from low PE families, higher media use has a less pronounced effect on language skills than in children from medium/high PE families. In particular, the effect of education is leveled out or virtually reversed when the duration of media use is >2 h. The association between higher media use and language difficulties in better-off children has been described by Ennemoser et al. [17]. A possible explanation is the ‘SES-mainstreaming hypothesis’ that describes the flattening effects of high media use for children from families with a different SES. On the one hand, this means decreasing language abilities for children from medium/high PE families with increasing media use. Our data confirm that part of the hypothesis. On the other hand, in the case of low PE, higher media use may—depending on the content—even be helpful for children who do not receive plenty of developmental support at home. Like Ennemoser et al. [17] found, our data do not maintain that part of the hypothesis.

It should be noted, however, that children from low PE families still show more linguistic problems overall. This is in line with results from, e.g., the National Education Panel [23], which examined the effect of PE on the developmental status of four- and five-year-old children. Children from low SES families clearly trailed in their competences compared to children from high SES families. Especially in the field of weaker language achievement, PE had a major effect.

### Limitations

Using the data of a complete school entry examination cohort, a social differential analysis has been possible. The cohort is representative for preschool children and there is no selection bias. However, since our data are based on a cross-sectional study and retrospective analysis, only associations can be shown but no causalities. The hypothesized association could be vice versa: increased media use can cause developmental deficits [36] but can also be the consequence of developmental problems [37]. However, the dose–response relationship of media use and language difficulties could be interpreted as an indicator of causality [38]. Moreover, daily media use may be underestimated since the information was given by the parents and could hence be influenced by social desirability. In the present study, the media used were not considered in a differentiated manner. Since detailed data about the type and content of the media used were not provided, we focused on the children’s daily media use as our independent variable. For further and more differentiated analyses, a differentiation into language-promoting (mainly audio media) and less language-promoting (mainly (audio-)visual media) would be necessary. Also, to observe incident developmental deficits, we excluded linguistically conspicuous children. But, by excluding them, it is possible that the effects of media use duration may be less salient.

## 5. Conclusions

The association between preschool media use and language difficulties at school entry depends on the examined aspects of language and on the social background of the child. Extensive media use is associated with language difficulties for all children. Given the progressive digitalization in recent years, media competence for young families remains important.

In addition, it is eminently important to repeatedly point out the impact of spoken language on children’s language acquisition. This concerns, e.g., the motherese, in which maternal language in everyday interaction with the child simultaneously has a promoting and correcting function. This approach belongs to implicit language education strategies and to a language-promoting behavior in the context of language education integrated into everyday life. It allows children to recognize and learn the grammatical rules of language. This can also serve as a kind of model or template for second-language acquisition for children with non-German FL, e.g., in daycare centers. It includes, i.a., corrective feedback, expanding or reshaping the child’s linguistic expressions, and establishing and maintaining a shared focus of attention between the caregiver and child (as described, e.g., in the Hamburg educational recommendations for children in daycare facilities on the educational area of communication [39]).

## Figures and Tables

**Table 1:** Sample description: observations (n) and percentages (%).

	First Language German		First Language Non-German		Medium/High Education		Low Education		Total	
	n	%	n	%	n	%	n	%	n	%
Gender										
Male	979	54	249	56	1056	54	172	57	1228	54
Female	838	46	194	44	903	46	129	43	1032	46
Daily media use										
0-<30 minutes	593	33	75	17	629	32	39	13	668	30
30-<60 minutes	845	47	165	37	909	46	101	34	1010	45
1-2 hours	311	17	122	28	327	17	106	35	433	19
>2 hours	68	3.7	81	18	94	4.8	55	18	149	6.6
Overall finding in language development										
No	1512	83	347	78	1621	83	238	79	1859	82
Yes	305	17	96	22	338	17	63	21	401	18
Finding in subtest pseudowords										
No	1616	89	397	90	1756	90	257	85	2013	89
Yes	201	11	46	10	203	10	44	15	247	11
Finding in subtest articulation										
No	1506	83	371	84	1632	83	245	81	1877	83
Yes	311	17	72	16	327	17	56	19	383	17
Finding in subtest plural formation										
No	1767	97	320	72	1855	95	232	77	2087	92
Yes	50	2.8	123	28	104	5.3	69	23	173	7.7
Finding in subtest preposition										
No	1767	97	312	70	1847	94	232	77	2079	92
Yes	50	2.8	131	30	112	5.7	69	23	181	8.0
Recommendation of language training										
No	1793	99	336	76	1884	96	245	81	2129	94
Yes	24	1.3	107	24	75	3.8	56	19	131	5.8
Total	1817	100.0	443	100.0	1959	100.0	301	100.0	2260	100.0

## Data Availability

Restrictions apply to the availability of these data. Data was obtained from municipal health authorities Rhein-Kreis Neuss and are available from SW with the permission of the municipal health authorities.
